# Pattern of Clinical Presentation in Patients With Lymphoma

**DOI:** 10.7759/cureus.62211

**Published:** 2024-06-12

**Authors:** Ahmadullah, Muhammad Bilal, Yaseen Khan

**Affiliations:** 1 Department of Medicine, Lady Reading Hospital, Peshawar, PAK; 2 Department of Internal Medicine, Lady Reading Hospital, Peshawar, PAK

**Keywords:** symptoms, signs, non-hodgkin lymphoma, hodgkin lymphoma, lymphoma

## Abstract

Introduction

Lymphomas take place when cells of the lymphatic system divide and re-divide in an uncontrolled fashion, and lymphomas have been termed as a “diverse group” of cancer, playing a major role in the area of oncology. The clinical behavior and manifestations of lymphomas in the head and neck region usually lack specific characteristics that would enable attribution to a specific lymphoma entity without biopsy and histological evidence. This study aimed to determine the frequency of common clinical features among patients with lymphoma.

Methods

This descriptive cross-sectional study was conducted at the Department of General Medicine, Lady Reading Hospital, Peshawar, from October 28, 2021 to April 28, 2022. The inclusion criteria consisted of individuals who were recently diagnosed with lymphoma and were between the ages of 10 and 50, regardless of their gender. This study enrolled a total of 186 patients diagnosed with lymphoma and assessed for common signs and symptoms. The data-gathering process included in-depth interviews, evaluations of medical history, physical exams, and initial investigations. The data analysis was done using IBM SPSS Statistics for Windows, Version 20.0 (Released 2011; IBM Corp., Armonk, New York, United States).

Results

The mean age of the patients was 34.5±9.6 years. Out of the total, 115 (61.8%) were men and 71 (38.2%) were women. With regard to symptoms, 134 (72%) had a fever, 80 (43%) had abdominal pain, 102 (54.8%) had vomiting, 49 (26.3%) had a headache, 111 (59.7%) had weight loss, and 17 (9.1%) had a cough. With regard to signs, 33 (17.7%) had painless lymphadenopathy, 58 (31.2%) had jaundice, 157 (84.4%) had anemia, 147 (79%) had hepatomegaly, 160 (86%) had splenomegaly, 24 (12.9%) had ascites, and 16 (8.6%) had abdominal tenderness.

Conclusion

The varied clinical appearance of lymphoma makes treatment difficult. In severe cases of lymphoma, early and timely diagnosis is crucial for proper and prompt treatment. The signs and symptoms, along with demographic information of patients, thorough medical history, imaging testing, and clinical examination, may indicate lymphoma.

## Introduction

Lymphomas include a wide range of malignancies that arise from the excessive proliferation of cells in the lymphatic system [1]. They have a prominent impact in the field of oncology [2]. These malignancies consist of many types of tumors that originate from lymphoid cells, which may be classified into B-cell, T-cell, and natural killer (NK)-cell lymphomas, as well as Hodgkin lymphoma (HL) and non-Hodgkin lymphoma (NHL) [3]. Approximately 90 subtypes and core kinds of lymphoma are classified by the World Health Organization [4]. Globally, the prevalence of different forms of lymphoma varies. NHL makes up around 90% of cases, whereas HL accounts for the remaining 10% [5]. Although there have been significant improvements in comprehending the molecular factors that contribute to the formation of lymphatic tissue, lymphoma has traditionally been approached as a singular ailment rather than a varied collection of separate abnormal growths [6].

Head and neck lymphoma should be diagnosed through histopathological examination, which involves analyzing tissue samples obtained via biopsy, due to the lack of specific clinical signs [7]. Typical symptoms include painless swelling of the lymph nodes, fatigue, systemic symptoms such as fever, excessive night sweats, unintentional weight loss, increased susceptibility to infections, and alterations in blood test results [8,9]. However, it is important to note that blood counts alone are insufficient for differentiating between indolent and aggressive lymphomas, except in cases where the lymphoma has progressed to a leukemic phase [10].

Previous research has identified a range of symptoms associated with lymphoma, such as asymptomatic lymphadenopathy, fever, night sweats, weight loss, cough, and itching. Comparative studies on the clinical manifestations of NHL and Hodgkin's disease (HD) reveal varying incidences of symptoms such as fever, weight loss, localized swellings, stomach complaints, and other markers [10]. This comparison highlights the distinct clinical presentations between different types of lymphoma, emphasizing the necessity for a thorough and nuanced approach to diagnosis and treatment.

The distinction between NHL and HD in terms of clinical manifestations is remarkable. The prevalence of fever was 67.3% in instances of NHL and 90.9% in patients with HD [11]. Weight loss was seen in 24.8% of instances of NHL, but a greater incidence of 81.8% was found in HD cases. Swollen areas in the neck, armpit, or groin were seen in 61.2% of NHL cases and in 90.9% of HD cases. The prevalence of abdominal discomfort was 55.1% in NHL patients compared to 27.2% in HD cases. Vomiting occurred in 30.6% of NHL cases and 45.4% of HD cases [7]. The incidence of headaches was 18.3% in NHL individuals and 18.1% in HD cases. The prevalence of joint aches was much lower in NHL (4.0%) compared to HD (45.4%). Additionally, 36.7% of NHL patients and 90.9% of HD cases had B symptoms. Lymphadenopathy was clinically seen in 59.1% of NHL patients and 90.9% of HD cases. Nevertheless, upon more thorough examination, the percentages escalated to 81.6% for NHL and reached a full 100% for HD [12].

The research aimed to provide insights into the frequency of typical clinical characteristics among lymphoma patients in the local area. The results will be disseminated among healthcare experts to enhance their understanding of the gravity of the problem and assist in formulating future research and treatment approaches.

## Materials and methods

This cross-sectional study was carried out at the Department of General Medicine, Lady Reading Hospital, Peshawar, from October 28, 2020, to April 28, 2021, for a duration of six months. The sample size was calculated to be 186, based on 14% of patients with a cough in lymphoma cases, a confidence level of 95%, and an absolute precision of 5%. A non-probability consecutive sampling technique was employed for patient recruitment.

All diagnosed lymphoma patients aged 10-50 years, irrespective of gender, were included in the study. Patients who had received any therapy prior to the study were excluded. Ethical approval was obtained from Lady Reading Hospital, and informed consent was secured from all patients or their guardians prior to participation.

Data collection was standardized to ensure methodological rigor and consistency across all participants. A structured questionnaire was designed and used to collect data. In-depth interviews were conducted to obtain comprehensive medical histories, and physical examinations were performed. These procedures were uniformly applied and conducted by an expert oncologist to minimize variations and reduce bias.

The collected data were entered into Microsoft Excel 2020 and subsequently coded for analysis using IBM SPSS Statistics for Windows, Version 20.0 (Released 2011; IBM Corp., Armonk, New York, United States). Descriptive statistics, including mean and standard deviation, were calculated for numerical variables such as age. Frequencies and percentages were computed for categorical variables, including gender and clinical features such as fever, lymphadenopathy, vomiting, and pain.

To determine the clinical features, the data were classified into groups based on age and gender. The chi-square test was applied to identify statistically significant differences, with a p-value <0.05 considered significant. The results were presented in tabular form to facilitate a clear and concise interpretation.

## Results

A total of 186 patients were recruited for the study, and all patients were categorized into different groups. Among the total, 69 (37.1%) were in the age group 31-40 years, followed by 57 (30.6%) in the age group >40 years, and 60 (32.3%) in the age group 20-30 years. Out of the total, 38.2% were male and 61.8% were female. The proportions of patients with fever, stomach discomfort, weight loss, and vomiting were found 134 (72%), 80 (43%), 111 (59.7%), and 102 (54.8%), respectively.

In contrast, the individuals evaluated exhibited a decreased incidence of symptoms such as cough (17, 9.1%), headache (49, 26.3%), and painless lymphadenopathy (33, 17.7%). In addition, it is worth noting that there is a higher occurrence of certain clinical manifestations in lymphoma cases. These include anemia (157, 84.4%), splenomegaly (160, 86.0%), and hepatomegaly (147, 79.0%). On the other hand, conditions such as jaundice (58, 31.2%), ascites (24, 12.9%), and abdominal tenderness (16, 8.6%) are relatively less common in the observed lymphoma cases (Table 1).

**Table 1:** Distribution of variables among lymphoma patients. The data have been represented as N and %.

Variables		Frequency (N)	Percentage
Age groups	20-30 years	60	32.3
	31-40 years	69	37.1
	>40 years	57	30.6
Gender	Male	115	61.8
	Female	71	38.2
Fever	Yes	134	72.0
	No	52	28.0
Abdominal pain	Yes	80	43.0
	No	106	57.0
Vomiting	Yes	102	54.8
	No	84	45.2
Headache	Yes	49	26.3
	No	137	73.7
Weight loss	Yes	111	59.7
	No	75	40.3
Cough	Yes	17	9.1
	No	169	90.9
Painless lymphadenopathy	Yes	33	17.7
	No	153	82.3
Jaundice	Yes	58	31.2
	No	128	68.8
Anemia	Yes	157	84.4
	No	29	15.6
Hepatomegaly	Yes	147	79.0
	No	39	21.0
Splenomegaly	Yes	160	86.0
	No	26	14.0
Ascites	Yes	24	12.9
	No	162	87.1
Abdominal tenderness	Yes	16	8.6
	No	170	91.4

The distribution of symptoms among lymphoma patients, examining variances based on age and gender, along with the relevant p-values, is presented as follows. Fever shows a gradual rise with age, but this tendency is not statistically significant (p=0.066). However, there is a notable discrepancy (p=0.001) in the occurrence of abdominal discomfort across different age groups, with much lower proportions seen in older age brackets. When examining the distribution of fever based on gender, significant disparities are observed (p=0.049), with a greater proportion of men reporting fever compared to women. In contrast, symptoms such as vomiting, headache, weight loss, and cough do not show substantial differences across genders (Table 2).

**Table 2:** Distribution of symptoms among lymphoma patients across age groups and gender. The data have been represented as N (%). *p<0.05 is considered significant.

Variables		Age groups (years)			p-value	Gender		p-value
		20-30	31-40	>40		Male	Female	
Fever	Yes	37 (61.7)	51 (73.9)	46 (80.7)	0.06	77 (67.0)	57 (80.3)	0.04*
	No	23 (38.3)	18 (26.1)	11 (19.3)		38 (33.0)	14 (19.7)	
Abdominal pain	Yes	35 (58.3)	31 (44.9)	14 (24.6)	<0.01*	53 (46.1)	27 (38.0)	0.28
	No	25 (41.7)	38 (55.1)	43 (75.4)		62 (53.9)	44 (62.0)	
Vomiting	Yes	27 (45.0)	38 (55.1)	37 (64.9)	0.09	62 (53.9)	40 (56.3)	0.74
	No	33 (55.0)	31 (44.9)	20 (35.1)		53 (46.1)	31 (43.7)	
Headache	Yes	18 (30.0)	15 (21.7)	16 (28.1)	0.53	32 (27.8)	17 (23.9)	0.59
	No	42 (70.0)	54 (78.3)	41 (71.9)		83 (72.2)	54 (76.1)	
Weight loss	Yes	39 (65.0)	38 (55.1)	34 (59.6)	0.51	74 (64.3)	37 (52.1)	0.09
	No	21 (35.0)	31 (44.9)	23 (40.4)		41 (35.7)	34 (47.9)	
Cough	Yes	2 (3.30)	8 (11.6)	7 (12.3)	0.16	14 (12.2)	3 (4.2)	0.06
	No	58 (96.7)	61 (88.4)	50 (87.7)		101 (87.8)	68 (95.8)	

The distribution of distinct clinical characteristics among patients with lymphoma, analyzing differences across various age groups and genders, along with the corresponding p-values. Within the age-wise distributions, certain clinical characteristics exhibit diverse patterns across distinct age groups. There are no substantial variations in the occurrence of painless lymphadenopathy, jaundice, anemia, ascites, and abdominal soreness across different age groups. Nevertheless, there are notable differences in the occurrence of hepatomegaly (p=0.017) and splenomegaly (p=0.017) across different age groups. The prevalence rates are greater in the younger age group (20-30 years) compared to the older age groups. When it comes to the distribution of clinical features based on gender, none of the analyzed characteristics show any statistically significant variations between men and girls. The incidence rates of features such as painless lymphadenopathy, jaundice, anemia, hepatomegaly, splenomegaly, ascites, and abdominal soreness are equal across genders (Table 3).

**Table 3:** Distribution of specific clinical features among lymphoma patients across age and gender. The data have been represented as N (%). *p<0.05 is considered significant.

Variables		Age groups (years)			p-value	Gender		p-value
		20-30	31-40	> 40		Male	Female	
Painless lymphadenopathy	Yes	12 (20.0)	11 (15.9)	10 (17.5)	0.83	21 (18.3)	12 (16.9)	0.81
	No	48 (80.0)	58 (84.1)	47 (82.5)		94 (81.7)	59 (83.1)	
Jaundice	Yes	18 (30.0)	27 (39.1)	13 (22.8)	0.14	40 (34.8)	18 (25.4)	0.17
	No	42 (70.0)	42 (60.9)	44 (77.2)		75 (65.2)	53 (74.6)	
Anemia	Yes	52 (86.7)	58 (84.1)	47 (82.5)	0.81	98 (85.2)	59 (83.1)	0.89
	No	8 (13.3)	11 (15.9)	10 (17.5)		17 (14.8)	12 (16.9)	
Hepatomegaly	Yes	49 (81.7)	60 (87.0)	38 (66.7)	0.01*	94 (81.7)	53 (74.6)	0.24
	No	11 (18.3)	9 (13.0)	19 (33.3)		21 (18.3)	18 (25.4)	
Splenomegaly	Yes	56 (93.3)	53 (76.8)	51 (89.5)	0.01*	100 (87.0)	60 (84.5)	0.64
	No	4 (6.7)	16 (23.2)	6 (10.5)		15 (13.0)	11 (15.5)	
Ascites	Yes	9 (15.0)	7 (10.1)	8 (14.0)	0.69	16 (13.9)	8 (11.3)	0.60
	No	51 (85.0)	62 (89.9)	49 (86.0)		99 (86.1)	63 (88.7)	
Abdominal tenderness	Yes	6 (10.0)	5 (7.2)	5 (8.8)	0.85	11 (9.6)	5 (7.0)	0.55
	No	54 (90.0)	64 (92.8)	52 (91.2)		104 (90.4)	66 (93.0)	

## Discussion

Our study corroborates previous reports on the diverse clinical manifestations of lymphomas, emphasizing the significance of accurate diagnosis and prompt treatment. Lymphomas, encompassing HL and NHL, exhibit varied clinical behaviors and anatomical distributions [13-17]. Previous classifications, such as the Revised European-American Classification of Lymphoid Neoplasm (REAL) and the WHO classifications, have evolved to integrate morphological, clinical, immunophenotypic, and genetic factors, aiding in precise diagnosis and prognosis determination [13-15].

Notably, our study underscores the diagnostic challenges posed by lymphomas in the head and neck regions. While extranodal presentations are common in NHL, distinguishing between different lymphoma entities solely based on clinical features remains elusive [18-20]. Histopathological assessment remains paramount for accurate diagnosis and timely initiation of therapy, especially given the potential consequences of delayed diagnosis on patient outcomes.

Advanced imaging modalities such as ultrasonography, computed tomography (CT), magnetic resonance imaging (MRI), and positron emission tomography (PET) offer valuable insights into lymphoma localization but may not reliably distinguish between NHL and HL [19-22]. Misinterpretation of radiological findings, coupled with clinical presentation, could lead to diagnostic delays and unfavorable prognoses [23-30].

Our study findings align with existing literature regarding demographic trends, symptomatology, and diagnostic challenges in lymphoma. Notably, the incidence of lymphoma among women has approached parity with men, possibly attributed to aging and immunocompromised states [31]. Socioeconomic factors and environmental variables also influence lymphoma incidence patterns [32,33].

Importantly, our findings highlight the necessity of cytological and histological examinations for lymphoma diagnosis, despite advancements in diagnostic technology. Lymphadenopathy, while a frequent indicator, requires careful evaluation to differentiate between benign and malignant etiologies [34-36]. The risk of misdiagnosis underscores the need for comprehensive diagnostic approaches, minimizing unnecessary treatments and interventions [37-40].

The limitations of this study include the relatively small sample size and the single-center design, which may not fully represent the broader population. The cross-sectional nature of the study restricts the ability to establish causal relationships between symptoms and lymphoma subtypes. Additionally, the reliance on self-reported symptoms and medical history could introduce recall bias, and the exclusion of patients who had received prior therapy may limit the generalizability of the findings to newly diagnosed individuals only.

Overall, this study contributes to the existing body of knowledge on lymphomas, emphasizing the importance of multidisciplinary approaches, including histopathological assessment, in achieving accurate diagnoses and optimizing patient outcomes. Future research directions should emphasize the integration of molecular abnormalities with clinical features to provide a more comprehensive understanding of lymphoma. Prospective studies should be designed to collect molecular data, such as genomic, transcriptomic, and proteomic profiles, alongside detailed clinical presentations from the onset of diagnosis through the course of treatment. By correlating molecular signatures with clinical characteristics such as age, sex, symptomatology, disease stage, and response to treatment, researchers can identify biomarkers that predict disease progression, therapeutic response, and patient outcomes. Such integrative studies could lead to the development of personalized treatment strategies, enhance prognostic accuracy, and potentially uncover novel therapeutic targets. Moreover, longitudinal data collection would enable the tracking of molecular changes over time, providing insights into disease dynamics and resistance mechanisms. Ultimately, this approach aims to bridge the gap between clinical practice and molecular biology, fostering a more holistic and precise approach to lymphoma management.

## Conclusions

The study highlights the diverse clinical presentation of lymphoma, which complicates timely diagnosis and treatment. The most common symptoms observed among the patients were fever, abdominal pain, vomiting, and weight loss, while signs such as anemia, splenomegaly, and hepatomegaly were prevalent. Notably, the distribution of these symptoms and signs varied across different age groups and between genders. Early and accurate diagnosis through a combination of thorough medical history, physical examination, imaging, and histological evidence is crucial for effective management and treatment of lymphoma. The findings underscore the importance of heightened awareness and a multidisciplinary approach among healthcare professionals to improve patient outcomes.
